# Clinical features and associated factors of coexisting intracerebral hemorrhage in patients with cerebral small vessel disease: a cross-sectional study

**DOI:** 10.1038/s41598-024-55968-9

**Published:** 2024-03-07

**Authors:** Yuan Gao, Ce Zong, Hongbing Liu, Ke Zhang, Hongxun Yang, Yunchao Wang, Yusheng Li, Bo Song, Yuming Xu

**Affiliations:** Department of Neurology, The First Affiliated Hospital of Zhengzhou University, No. 1 Eastern Jianshe Road, Erqi District, Zhengzhou, 450052 Henan Province China

**Keywords:** Intracerebral hemorrhage, Cerebral small vessel disease, Calcium, Phosphorus, Neuroscience, Biomarkers, Neurology, Risk factors

## Abstract

Intracerebral hemorrhage (ICH) is generally considered to be closely related to cerebral small vessel disease (CSVD), leading to a poor prognosis. However, the coexistence of ICH in general CSVD patients and related factors remain underreported. In our cross-sectional study, we screened 414 CSVD patients from a database at the Department of Neurology, First Affiliated Hospital of Zhengzhou University (September 2018 to April 2022). Imaging biomarkers of CSVD and coexisting ICH lesion were assessed. Factors associated with coexisting ICH in CSVD were determined using multivariate logistic regression analysis. ICH was observed in 59 patients (14.3%). Multivariate logistic regression showed that previous history of ischemic stroke or transient ischemic attack (OR 5.189, 95%CI 2.572–10.467, *P* < 0.001), high-grade perivascular space in the basal ganglia (n > 10) (OR 2.051, 95%CI 1.044–4.027, *P* = 0.037) and low adjusted calcium-phosphorus product (OR 0.728 per 1 [mmol/L]^2^ increase, 95%CI 0.531–0.998, *P* = 0.049) were associated with coexisting ICH in CSVD patients. The considerable proportion of coexisting ICH and revelation of associated factors in general CSVD patients alert physicians of the potential risk of the reoccurrence of ICH, and might have a significant impact on therapeutic strategies.

## Introduction

Cerebral small vessel disease (CSVD) refers to as a set of pathological damage involving the small arteries or veins of the brain, which exhibit various imaging features such as recent small subcortical infarcts (RSSIs), white matter hyperintensities (WMHs), cerebral microbleeds (CMBs), enlarged perivascular space (EPVS), lacunar, cortical superficial siderosis (cSS) and brain atrophy^1,2^. Intracerebral hemorrhage (ICH) was generally considered to be closely related to CSVD usually leading to a poor prognosis^3–5^. Previous research has indicated that patients with ICH frequently exhibit more pronounced CSVD imaging characteristics. And a high prevalence of coexisting ICH has also been observed, ranging between 10.1 and 25%, in certain subtypes of CSVD, like cerebral autosomal dominant arteriopathy with subcortical infarcts and leukoencephalopathy (CADASIL) or small subcortical infarcts (SSIs)^6–8^. At the same time, a number of risk factors, such as infarct location, total CSVD burden, number and location of CMBs have been raised^8,9^. However, there is a scarcity of data on the prevalence and associated factors of coexisting ICH in a more general CSVD population defined by imaging criteria. Identifying patients at a higher risk of ICH is crucial to mitigate potential adverse effects of therapeutic strategies. Therefore, this cross-sectional study aims to investigate the proportion, imaging features and associated factors of coexisting ICH in a general CSVD population defined by imaging criteria.

## Materials and methods

### Patient selection

The patients were screened from a database in the Department of Neurology in the First Affiliated Hospital of Zhengzhou University from September 2018 to April 2022. These patients were from the neurology wards or outpatient clinics, admitted with symptoms of acute cerebral infarction, chronic cognitive impairment, or incidentally detected asymptomatic lacunar, etc., and they were improved with standardized tests and examinations, screened according to the inclusion and exclusion criteria, and then finally checked again by the specialized senior neurologists.

In the database, CSVD patients were enrolled by classical CSVD imaging biomarkers. Inclusion criteria was as follow: (a) age ≥ 18 years. (b) visible CSVD lesions on brain magnetic resonance imaging (MRI), satisfying one of the following items: WMHs with Fazekas score ≥ 2 or Fazekas = 1 and ≥ 2 vascular risk factors (hypertension, hyperlipidemia, diabetes mellitus, obesity, current smoking, previous events of vascular origin other than stroke) or Fazekas = 1 and combined lacunar foci; imaging suggestive of RSSIs; lacunar foci of possible vascular origin; EPVS, CMBs in the brain. (c) independence of daily life (modified Rankin Scale ≤ 2). Exclusion criteria: (a) ischemic lesions in the cortex or with maximum axial diameters > 20 mm on brain MRI; (b) dementia caused by brain damage due to diagnosed neurodegenerative diseases (e.g., Alzheimer's disease, Parkinson's disease); (c) WMHs defined of non-vascular origin, such as multiple sclerosis, adult cerebral white matter dysplasia, metabolic encephalopathy, etc.; (d) history of cerebrovascular malformation/cerebral aneurysmal subarachnoid hemorrhage, or untreated aneurysm (> 3 mm in diameter); (e) psychiatric disorders diagnosed according to the DSM-V diagnostic criteria; (f) contraindications to MRI examination (e.g., claustrophobia, etc.); (g) intracranial occupancy, poisoning, metabolic or infection, demyelination-related diseases. (h) suffering from serious organic diseases, such as malignant tumors, with an expected survival time of < 5 years. In addition, in our study, those patients with incomplete hematological data and imaging information (T1 and T2-weighted MRI, diffusion-weighted imaging [DWI], magnetic resonance angiography [MRA], fluid-attenuated inversion recovery [FLAIR], susceptibility-weighted imaging [SWI], etc.) were further excluded. Our study was approved by the Ethics Committee of the First Affiliated Hospital of Zhengzhou University (Ethics Review Number: 2021-KY-1059-002). All patients signed informed consent.

### Data collection

We collected demographic indicators (age, gender, disease history, etc.), hematological indicators (glucose, lipids, electrolytes, liver and kidney function, etc.), and imaging parameters of CSVD. Hypertension defined as systolic blood pressure (SBP)/diastolic blood pressure (DBP) ≥ 140/90 mmHg or previous use of antihypertensive medication. Diabetes defined as fasting blood glucose > 7.0 mmol/L or glycated hemoglobin > 6.5% or history of glucose-lowering medication. Hyperlipidemia was considered as total cholesterol (TC) > 5 mmol/L or low-density lipoprotein (LDL) cholesterol > 3.62 mmol/L or prior treatment with lipid-lowering medication. History of cerebrovascular disease (CVD) was defined as previous ischemic stroke (IS) or transient ischemic attack (TIA). History of smoking was defined as current regular smoking or quitting within 6 months. History of alcohol use was defined as regular alcohol use currently or within 6 months of abstinence. Calcium and phosphorus metabolism (CPM) -related indicators include serum calcium (Ca), phosphorus (P) and calcium-phosphorus product (Ca * P). Serum Ca is adjusted if albumin < 35 g/L, or > 51 g/L, and adjusted Ca (mmol/L) = Ca (mmol/L) + 0.02 [40 − albumin (g/L)]^10^.

### Image evaluation

All patients underwent preoperative examination using Siemens 3.0T (Skyra, Verio, Prisima) superconducting MRI. The scanning sequences and parameters are as follows: (a) Transverse and sagittal T1WI sequence with a slice thickness of 5 mm and echo time (TE) of 2.5 ms; (b) Transverse T2WI sequence with a slice thickness of 5 mm and TE of 2.5 ms; (c) Transverse T2 FLAIR sequence with a slice thickness of 5 mm, repetition time (TR) of 6500 ms, and TE of 85 ms; (d) Transverse DWI sequence with a slice thickness of 5 mm; TR of 4600 ms, TE of 80 ms, b values of 0 and 1000 s/mm^2^, automatic reconstruction of ADC map after scanning; (e) Conventional enhancement of MRI with a slice thickness of 5 mm and TE of 2.5 ms; (f) SWI sequence with TR/TE = 29 ms/20 ms, layer thickness of 0.6 mm, spatial resolution of 0.2 mm × 0.2 mm × 0.6 mm, FOV of 6 cm × 6 cm, flip angle of 15 degrees, and matrix of 256 × 256.

Coexisting ICH were defined as iron-containing hemoglobin deposits of more than 10 mm in diameter on SWI regardless of history of ICH and whether it was clinically symptomatic or not^8^. The location (cortex, deep, and subtentorial) and number of ICH lesions were evaluated. Cortical superficial siderosis (cSS) refers to linear deposits formed by the confinement of iron-containing hemoflavin, a blood breakdown product, to the cortical sulcus of the cerebral hemispheric bulge, which shows a loss of signal (low intensity) on T2*-GRE or SWI sequences^11^. CSVD lesions visible on brain MRI, satisfying one of the following items^2,12^: (a) WMHs in the brain (scattered or diffuse lesions distributed in the subcortical white matter, periventricular and semi-oval center of the cerebral cortex, with irregular edges and high intensity on T2WI and FLAIR imaging); (b) imaging suggestive of RSSIs; (c) possibly lacunar foci of vascular origin (3–15 mm in diameter in the distribution area of the penetrating artery with cerebrospinal fluid signal); (d) EPVS (≤ 3 mm in diameter along the vascular alignment); (e) CMBs in the brain (low signal ≤ 10 mm in diameter on SWI, no high signal in the corresponding lesions on T1 and T2). We evaluated the following four imaging features: (1) High-grade white matter hyperintensities (HWMH) were defined as periventricular or paraventricular Fazekas scores ≥ 2^13^; (2) High-grade perivascular space (HPVS) were defined as the coexisting of more than 10 visible EPVS in the basal ganglia (BG)^14,15^; (3) lacunar was defined as the number of lacunar foci ≥ 1; (4) CMBs was defined as number of CMBs ≥ 1. Also, information of the location (cortex, deep and subtentorial) and number of CMBs was obtained and the number of CMBs was categorized by using 5 and 10 as cut-off values respectively. Meanwhile, we used a 0 to 4 scale to calculate the total CSVD burden, each of the above items was scored 1 point, and the items were summed to the total CSVD score. ICH assessment was conducted by two neurologists blinded to study design with more than 5 years of diagnostic experiences. After examining images from a group of 50 consecutive patients, the inter-rater reliabilities between the 2 investigators were 0.88 by κ statistics for ICH.

### Statistical analysis

Continuous and categorical variables were described by means ± standard deviation and proportions, respectively. For the comparative analysis of baseline characteristics, we applied the t-test or Mann–Whitney U-test for continuous variables, contingent on their distributional properties, and the chi-square test for categorical variables. All variables with *P* < 0.05 in the univariate analysis were included in the multivariate analysis. Multivariate logistic regression were employed to analyse the factors associated with coexisting ICH in CSVD patients. The statistical computations were performed using SPSS (version 26.0) and R (version 4.1.2). We established a threshold for statistical significance at a *p*-value of less than 0.05.

### Informed consent

All procedures carried out in studies involving human participants are consistent with the ethical standards of institutions and/or national research councils, as well as with the 1964 Helsinki Declaration and its subsequent amendments or similar ethical standards. Our study was approved by the Ethics Committee of the first Affiliated Hospital of Zhengzhou University (Ethics Review Number: 2021-KY-1059-002). All patients signed informed consent.

## Results

### Baseline

A total of 414 out of 732 CSVD patients were included in the final analysis after excluding those with missing necessary imaging data (n = 262) and clinical information (n = 56). Comparison of clinical characteristics between included and excluded data were detailed in Table S1. Results showed no significant differences in demographic data, but some hematologic measures (e.g., white cell count, platelet count, and fasting plasma glucose). Of those patients ultimately included in the analysis, The mean age was 62.5 ± 10.4 years old, 153 patients (37.0%) was female. Additional detailed demographic information was presented in Table 1.

**Table 1:** Comparison of baseline characteristics in CSVD patients with intracerebral hemorrhage and without.

Variables	ALL	without intracerebral hemorrhage	with intracerebral hemorrhage	*p*. overall
	(N = 414)	(N = 355)	(N = 59)	
**Demographic information**				
Female, n (%)	153 (37.0%)	133 (37.4%)	20 (33.9%)	0.712
Age, year^#^	62.5 (10.4)	62.6 (10.4)	61.4 (10.5)	0.411
SBP, mmHg^#^	141 (18.3)	141 (18.4)	140 (18.2)	0.718
DBP, mmHg^#^	83.8 (11.7)	84.2 (11.7)	80.9 (11.4)	**0.046**
Hypertension, n (%)	333 (80.4%)	282 (79.4%)	51 (86.4%)	0.281
CHD, n (%)	43 (10.4%)	38 (10.7%)	5 (8.47%)	0.816
Diabetes, n (%)	156 (37.7%)	136 (38.3%)	20 (33.9%)	0.615
IS/TIA, n (%)	161 (39.3%)	121 (34.3%)	40 (70.2%)	**< 0.001**
Hyperlipidemia, n (%)	199 (48.5%)	169 (48.1%)	30 (50.8%)	0.808
Smoking, n (%)	114 (27.5%)	98 (27.6%)	16 (27.1%)	0.938
Drinking, n (%)	104 (25.1%)	92 (25.9%)	12 (20.3%)	0.472
**Laboratory data**				
WBC, x10^9^L^#^	6.70 (2.17)	6.62 (2.02)	7.20 (2.88)	0.148
Lymphocyte, x10^9^L^#^	1.80 (1.14)	1.76 (0.58)	2.03 (2.67)	0.447
Neutrophil, x10^9^L^#^	4.31 (2.02)	4.23 (1.87)	4.84 (2.72)	0.105
PLT, x10^9^L^#^	215 (63.5)	214 (62.1)	221 (71.5)	0.520
ALB, g/L^#^	41.6 (3.94)	41.7 (3.92)	41.2 (4.07)	0.382
Fibrinogen, mg/dl^#^	2.97 (0.69)	2.97 (0.68)	2.98 (0.77)	0.907
FBG, mmol/L^#^	5.98 (2.21)	5.96 (2.19)	6.16 (2.30)	0.553
HbA1c, %^#^	6.45 (1.81)	6.47 (1.88)	6.36 (1.29)	0.589
Homocysteine, μmol/L^#^	16.4 (11.3)	16.4 (11.2)	16.4 (12.3)	0.991
TC, mmol/L^#^	3.98 (1.13)	4.02 (1.16)	3.74 (0.95)	0.053
TG, mmol/L^#^	1.45 (1.10)	1.47 (1.13)	1.35 (0.89)	0.361
HDL, mmol/L^#^	1.12 (0.32)	1.13 (0.33)	1.07 (0.28)	0.172
LDL, mmol/L^#^	2.45 (0.96)	2.48 (0.97)	2.26 (0.83)	0.086
eGFR, ml/min^#^	91.3 (16.4)	91.1 (16.1)	93.1 (18.2)	0.440
P, mmol/L^#^	1.14 (0.20)	1.15 (0.20)	1.10 (0.18)	0.070
Ca, mmol/L^#^	2.27 (0.11)	2.28 (0.11)	2.24 (0.11)	**0.044**
Adjusted Ca, mmol/L^#^	2.60 (0.50)	2.62 (0.51)	2.48 (0.47)	**0.044**
Ca * P, (mmol/L)^2#^	2.60 (0.51)	2.62 (0.51)	2.47 (0.47)	**0.035**
Adjusted Ca * P, (mmol/L)^2#^	3.07 (1.14)	3.11 (1.16)	2.80 (0.97)	**0.039**
**Imaging features**				
LI, n (%)	217 (52.4%)	183 (51.5%)	34 (57.6%)	0.469
Microbleeds, n (%)	248 (59.9%)	202 (56.9%)	46 (78.0%)	**0.004**
Number of CMBs > 5, n (%)	49(11.8%)	38(10.7%)	11(18.6%)	0.080
Number of CMBs > 10, n (%)	31(7.5%)	23(6.5%)	8(13.6%)	0.056
Location of microbleeds				
Cortex, n (%)	97 (23.4%)	77 (21.7%)	20 (33.9%)	0.060
Deep, n (%)	204 (49.3%)	168 (47.3%)	36 (61.0%)	0.071
Subtentorial, n (%)	82 (19.8%)	64 (18.0%)	18 (30.5%)	**0.040**
HWMH, n (%)	162 (39.1%)	131 (36.9%)	31 (52.5%)	**0.031**
Location of WMHs				
Paraventricular *	1.00 [1.00; 2.00]	1.00 [1.00; 2.00]	1.00 [1.00; 2.00]	0.092
Deep *	1.00 [0.00; 2.00]	1.00 [0.00; 2.00]	1.00 [0.00; 2.00]	0.309
Total *	2.00 [1.00; 4.00]	2.00 [1.00; 4.00]	2.00 [1.00; 4.00]	0.138
HPVS, n (%)	175 (42.3%)	139 (39.1%)	36 (61.0%)	**0.002**
Location of EPVS				
Centrum semiovale, n (%)	42 (10.1%)	35 (9.86%)	7 (11.9%)	0.811
Basal ganglia n (%)	371 (89.6%)	316 (89.0%)	55 (93.2%)	0.453
cSS, n (%)	17 (4.1%)	15 (4.2%)	2 (3.4%)	0.765
Total CSVD burden*	2.00 [1.00; 3.00]	2.00 [1.00; 3.00]	3.00 [2.00; 3.00]	**< 0.001**

*SBP* systolic blood pressure, *DBP* diastolic blood pressure, *CHD* coronary heart disease, *IS* ischemic stroke, *TIA* transient ischemic attack, *WBC* white blood cell, *PLT* platelet, *ALB* albumin, *FBG* fasting blood glucose, *HbA1c* glycation hemoglobin, *TC* total cholesterol, *TG* triglycerides, *LDL* low-density lipoprotein, *HDL* high-density lipoprotein, *eGFR* estimated glomerular filtration rate, *Ca* calcium, *P* phosphorus, Ca * P calcium-phosphorus product, *LI* lacunar infarction, *CMBs* cerebral microbleeds, *WMHs* white matter hyperintensities, *HWMH* high-grade white matter hyperintensities, *HPVS* high-grade perivascular space, *EPVS* enlarged perivascular space, *CSVD* cerebral small vessel disease, *cSS* cortical superficial siderosis.

^*^: median (IQR); ^#^: mean ± standard deviation.

Significant values are in bold.

### ICH and CMBs lesion in CSVD patients

**Figure 1:**
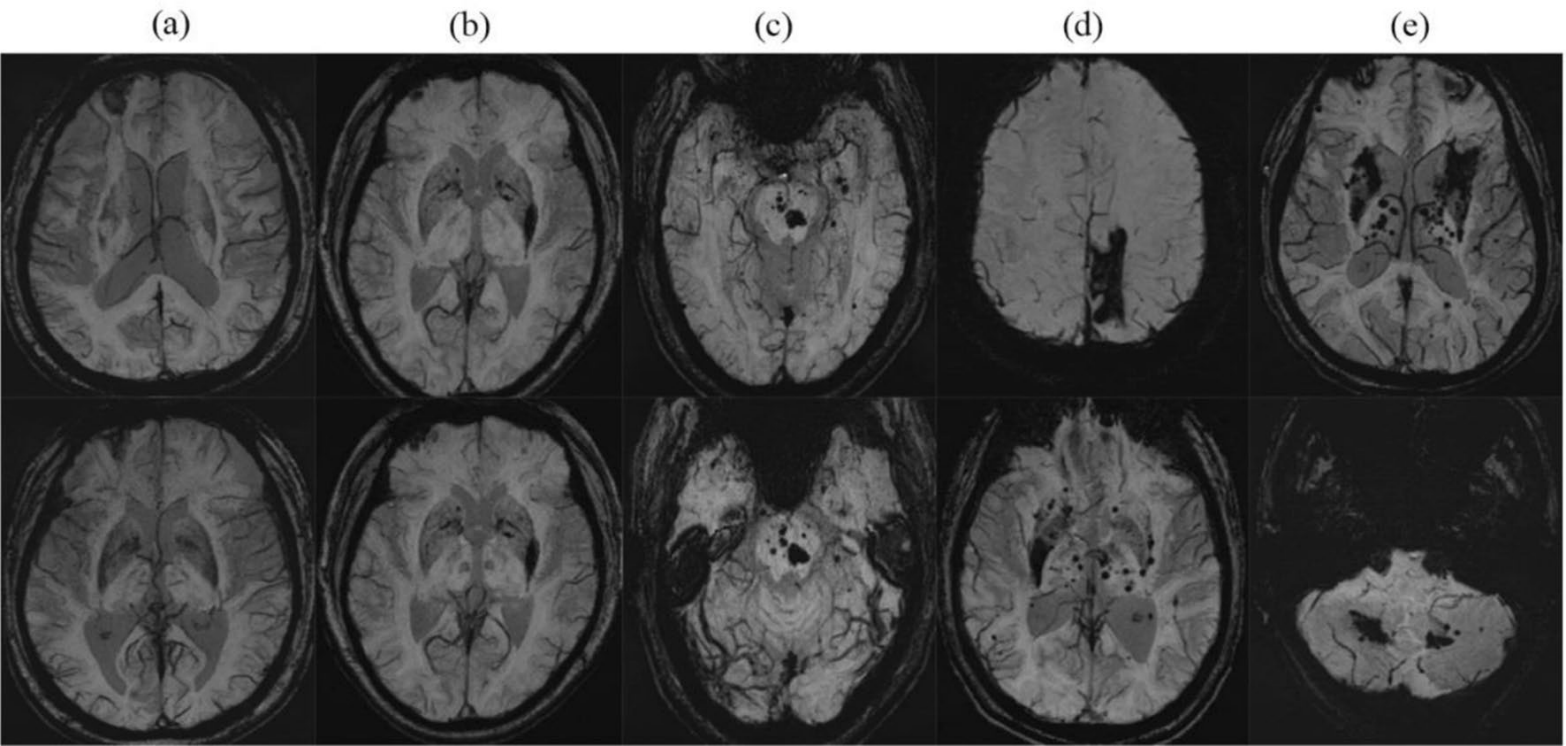
Five types of cerebral hemorrhage locations. (**a**) strictly in the cortex. (**b**) strictly in the deep area. (**c**) strictly in the subtentorial area. (**d**) in the cortex and deep area. (**e**) in the deep and subtentorial area.

ICH lesions were detected on SWI images in 59 patients (14.3%), of which 22 patients (37.2%) had a history of ICH. Information on the locations and numbers of ICH lesions were shown in Fig. S1 and S2. Of all 59 CSVD patients with coexisting ICH, 78% strictly located in deep area, 12% strictly in the cortex, 5% in both the cortex and deep region, 3% strictly in the brainstem and 2% in both the deep and cerebellum. On the other hand, single ICH lesions were detected on DWI in 39 patients, while 15 patients exhibited 2 ICH lesions, 4 patients had 3 ICH lesions, and only one patient presented with 4 ICH lesions. Figure 1 illustrates five types of ICH lesions, with a prevailing occurrence of strictly deep hemorrhages. Among all the patients, cSS was observed in 17 (4.1%) cases, with 2 (3.4%) cases presenting with ICH and 15 (4.2%) cases without. CMBs were presented in 248 patients (59.9%), among which 28 patients (11.3%) distributed exclusively in the cortex, 107 (43.1%) strictly in the deep area, 12 (4.8%) strictly in the subtentorial region, and 101 (40.8%) in mixed region. Among all CSVD patients, 23 (5.6%) met the criteria for probable cerebral amyloid angiopathy (CAA) as per the Boston Diagnostic Criteria 2.0^16^.

### Univariate logistic regression analysis on associated factors of ICH in CSVD patients

In univariate analysis, several significant differences were noted in CSVD patient with coexisting ICH compared to CSVD patient without, including lower DBP (*P* = 0.046), a higher proportion of IS/TIA history (*P* < 0.001), lower Ca level (*P* = 0.044), lower adjusted Ca level (*P* = 0.044), lower Ca * P (*P* = 0.035), lower adjusted Ca * P (*P* = 0.039), a higher prevalence of CMBs (*P* = 0.004), subtentorial microbleeds (*P* = 0.040), HWMHs (*P* = 0.031), HPVS(*P* = 0.002) and a higher total CSVD burden (*P* < 0.001) (Table 1). When categorizing the number of CMBs into > 0, > 5, > 10, only the category of CMBs > 0 showed a significant association with coexisting ICH (*P* = 0.004), while the category of CMBs > 5 (*P* = 0.080) and > 10 (*P* = 0.056) only showed a trend without statistical significance. However, the presence of cSS was not significantly different between the two groups (*P* = 0.765).

### Multivariate logistic regression analysis on associated factors of ICH in CSVD patients

The total CSVD burden and its subcomponents (HPVS, HWMHs, microbleeds, subtentorial microbleeds) were included separately in the multivariate analysis to make the results more stable. In the multivariate logistic regression analysis, we constructed four models based on CPM-related indicators (P, Ca * P, Ca, adjusted Ca, adjusted Ca * P). The results showed that IS/TIA (OR 4.991, 95%CI 2.500–9.962, *P* < 0.001), total CSVD burden (OR 1.350, 95%CI 1.034–1.762, *P* = 0.027), adjusted Ca * P (OR 0.730 per 1 [mmol/L]^2^ increase, 95%CI 0.543–0.998, *P* = 0.049) were associated with coexisting ICH in CSVD patients after adjusting for DBP. Further results remained significantly correlated even after adjusting for DBP, HWMH, CMBs and subtentorial microbleeds (IS/TIA: OR 5.189, 95%CI 2.572–10.467, *P* < 0.001; HPVS: OR 2.051, 95%CI 1.044–4.027, *P* = 0.037; adjusted Ca * P: OR 0.728 per 1 [mmol/L]^2^ increase, 95%CI 0.531–0.998, *P* = 0.049) (Table 2).

**Table 2:** Multivariate logistic regression analysis of intrracerebral hemorrhage in CSVD patients.

	Model 1			Model 2			Model 3			Model 4		
	Variables	Adjusted OR (95%CI)	*P*-value	Variables	Adjusted OR (95%CI)	*P*-value	Variables	Adjusted OR (95%CI)	*P*-value	Variables	Adjusted OR (95%CI)	*P*-value
	IS/TIA	4.734 (2.384–9.403)	< 0.001	IS/TIA	4.778 (2.410–9.471)	< 0.001	IS/TIA	4.983 (2.495–9.952)	< 0.001	IS/TIA	4.991 (2.500–9.962)	< 0.001
Step1*	Total CSVD burden	1.363 (1.045–1.779)	0.022	Total CSVD burden	1.335 (1.025–1.738)	0.032	Total CSVD burden	1.354 (1.037–1.768)	0.026	Total CSVD burden	1.350 (1.034–1.762)	0.027
	p		NE	p		NE	Ca * P	0.485 (0.248–0.951)	0.035	Adjusted Ca * P	0.730 (0.543–0.998)	0.049
	Ca	0.044 (0.002–0.856)	0.039	Adjusted Ca	0.507 (0.259–0.995)	0.048						
Step2^#^	IS/TIA	5.172 (2.614–10.234)	< 0.001	IS/TIA	5.470 (2.749–10.887)	< 0.001	IS/TIA	5.274 (2.667–10.430)	< 0.001	IS/TIA	5.189 (2.572–10.467)	< 0.001
	HPVS	2.430 (1.282–4.609)	0.007	HPVS	2.346 (1.230–4.473)	0.010	HPVS	2.257 (1.192–4.273)	0.012	HPVS	2.051 (1.044–4.027)	0.037
	p		NE	p		NE	Ca * P	0.497 (0.257–0.964)	0.039	Adjusted Ca * P	0.728 (0.531–0.998)	0.049
	Ca	0.034 (0.002–0.070)	0.029	Adjusted Ca	0.503 (0.259–0.978)	0.043						

Model 1–4: There was covariance between Ca, Adjusted Ca, Ca * P, Adjusted Ca * P, and thus it needed to be put into the model separately.

Step 1–2: There was covariance between CSVD total burden and HPVS, high-grade white matter hyperintensities, Microbleeds, Subtentorial, and therefore it needed to be corrected separately.

*IS* ischemic stroke, *TIA* transient ischemic attack, *Ca* calcium, *P* phosphorus, *Ca* * *P* calcium-phosphorus product, *HPVS* high-grade perivascular space, *CSVD* cerebral small vessel disease. *NE* not entry, *OR* odds ratio, *CI* confidence interval; *p* < 0.05 was considered meaningful.

*Adjusted for diastolic blood pressure, IS/TIA, total CSVD burden.

^#^Adjusted for diastolic blood pressure, IS/TIA, HPVS, high-grade white matter hyperintensities, Microbleeds, Subtentorial.

## Discussion

This cross-sectional study unveiled several novel insights : (a) The study was noted a 14.3% prevalence of coexisting ICH lesion detected on SWI within general CSVD patients; (b) Key associated factors identified for coexisting ICH in CSVD patients included reduced serum concentrations of Ca and Ca * P, previous history of IS/TIA, and high grade BG-EPVS (n > 10).

Previous studies have disclosed a higher prevalence of coexisting ICH, ranging from 10.1 to 25%, in certain CSVD types, such as CADASIL and SSIs. This finding has been further validated in our study in a broader CSVD population defined by classic imaging markers. The reason for the different proportions of ICH may be related to the fact that the majority in this study were hypertensive CSVD with a small proportion of cSS or CAA, thus small atherosclerosis/deep penetrating arterial disease may be more common in our study, and consequently the proportion of ICH may be lower than that previously reported. It underscores the close association between CSVD and ICH. Our research provides more interesting details on the imaging characteristics of coexisting ICH in CSVD patients. In our study, the distribution of strictly deep, strictly lobar and other types of ICH accounted for 78%, 12%, 10% respectively (as shown in Fig. 1, S1, and S2). Isolated deep ICH was the most prevalent, likely due to the fact that the majority of patients (80.4%) had hypertension. The distribution of CMBs were primarily strictly deep and mixed types (43.1%, 40.8% respectively), indicating hypertension as a common vascular pathology mechanism.

cSS was observed in 17 (4.1%) cases in our study. cSS was associated with symptomatic ICH^17^. Charidimou A et al. demonstrated that the risk of symptomatic ICH at 5-year follow-up in patients with cSS at baseline was 19% (95% CI 11–32%), which was higher than the 6% (95% CI 3–12%) in patients without cSS. And cSS was consistently correlated with an increased risk of future lobar ICH in CAA^18,19^. In addition, It should be noted that 5.6% of cases meet the diagnostic criteria for probable CAA according to the Boston criteria 2.0. Previous studies showed that CAA was associated with spontaneous ICH and a higher risk of ICH recurrence, especially lobar ICH^20,21^. These findings raise concerns about the increased future hemorrhagic stroke risk in CSVD patients, especially when combined with antithrombotic drug use. Studies showed that ICH patients have recurrence rates ranging from 1.3 to 7.4% annually and associated IS incidents from approximately 1% to 6%^22,23^. It is necessary to weigh the risks of bleeding versus ischemia. Due to the cross-sectional design, this question remains unanswered in our study, and needs future longitudinal studies. Given CAA’s heightened bleeding risk, there is a consensus that in CSVD patients with coexisting CAA, particularly the use of anticoagulants, should be avoided if possible. Hence, our study results remind clinicians to perform SWI examinations in CSVD patients to exclude potential CAA cases and mitigate risks.

In our study, previous history of IS/TIA was founded to be associated with the coexisting of ICH, which was consistent with previous studies^24,25^. This might be related to blood flow disturbance and blood–brain barrier instability caused by damage to local microvascular pericytes after the occurrence of cerebral infarction^26^. Furthermore, we observed a significant correlation between EPVS, particularly in the BG, and coexisting ICH. EPVS is commonly recognized as a sensitive imaging marker for CSVD, and numerous studies have affirmed its association with spontaneous ICH. For instance, Park et al. conducted a retrospective analysis of 150 patients with primary ICH and 271 age- and sex-matched controls, revealing more severe BG-EPVS in the ICH group^27^. Best et al. reported in a follow-up study involving 1386 patients with atrial fibrillation and recent TIA or IS that BG-EPVS may be a risk factor for anticoagulant-associated ICH^28^. The presence of EPVS in the BG may indicate the local blood pressure status of small arteries in deep penetrating branches, leading to damage to the blood–brain barrier or extravasation of perivascular fluid, ultimately contributing to ICH^28,29^. Notably, when considering individual imaging indices, it was observed that, although the coexistence of total CSVD burden was associated with the risk of ICH, EPVS showed a more significant correlation with ICH (Table 2)^30^. However, a recent study has not confirmed the correlation between EPVS in the BG region and the risk of ICH recurrence^31^. The contradictory results may require further exploration through future clinical studies with larger sample sizes.

Unlike previous investigations focusing on CPM-related indicators primarily within hospitalized populations with renal disease or haemodialysis^10,32–34^, Our study for the first time identified an association between CPM-related indicators and ICH within a CSVD cohort. Previously, Guo et al.^33^ identified an independent association between reduced serum Ca levels and haemorrhagic transformation (HT) after thrombolysis in a study involving 362 post-thrombolytic patients. Alberts et al.^35^ revealed a link between lower serum Ca concentrations and hematoma expansion. While, our study revealed that serum Ca concentration and Ca * P on admission were associated with coexisting ICH in CSVD patients. Although, the study design limitations prevent establishing a clear cause-and-effect relationship between the two, the underlying mechanisms may involve: (1) Local hypertension induced by the constriction of small arteries at low Ca levels. Previous studies have suggested an association between Ca^2+^ Sparks and the BK channel in regulating arterial diameter in the human brain^36^, potentially leading to local hypertension at low Ca levels and increased susceptibility to ICH; (2) coagulation disorders and bleeding tendency due to low Ca concentrations^33,35,37,38^. Calcium’s active role in the coagulation process, particularly in prothrombin formation and activity has been observed^39^, and hypocalcaemia, more commonly found in patients with coagulation dysfunction, may significantly prolong clotting time, thereby contributing to ICH occurrence^40,41^; (3) Disturbed calcium homeostasis. Ca^2+^ inward flow is implicated in neuronal cell death, and disturbances in calcium homeostasis elevate intracellular Ca^2+^, disrupting cell membrane integrity. This activation of membrane phospholipases and protein kinases may affect the blood–brain barrier, potentially causing ICH^42,43^. In our results, the lower Ca * P was associated with ICH while serum P levels were not significant, so we hypothesized that its relationship with ICH might be indirect due to an decrease in serum Ca levels leading to a subsequent decrease in the Ca * P^44^. While, we cannot rule out the possibility that this phenomenon may be secondary to ICH, pending further research for clarification in the future.

This study has some limitations. Firstly, this is a single-center study conducted within a hospital, a larger multi-center cohort study with larger samples is essential to validate our conclusions in the future; Secondly, our results can only establish a correlation between lower serum Ca concentration and Ca * P on admission and the risk of combined ICH in CSVD patients. However, they do not elucidate the cause-and-effect relationship between these variables, necessitating further prospective studies; Finally, although our study excluded some diseases that affect CPM such as metabolic nephropathy and thyroid disease, there might still be underlying pathological conditions impacting the accuracy of Ca and P concentrations and introducing some bias.

## Conclusion

Our study observed a 14.3% prevalence of coexisting ICH lesion detected on SWI within general CSVD patients. Associated factors for the coexistence of ICH in CSVD patients were identified, including reduced serum concentrations of Ca and Ca * P, previous history of IS/TIA, and high grade BG-EPVS (n > 10).

### Supplementary Information

Supplementary Information.

## Data Availability

The datasets used and/or analysed during the current study available from the corresponding author on reasonable request.
