# Supplementary material for: Clinical features and associated factors of coexisting intracerebral hemorrhage in patients with cerebral small vessel disease: a cross-sectional study
Source: Sci Rep. 2024 Mar 7;14:5596. doi: 10.1038/s41598-024-55968-9 (PMC10920749; doi:10.1038/s41598-024-55968-9)
Supplement: Supplementary file 1 — Supplementary Information. [file 41598_2024_55968_MOESM1_ESM.docx]

**Supplementary Materials**

**Full title**

**Clinical features and associated factors of coexisting intracerebral hemorrhage in patients with cerebral small vessel disease: a** **cross-sectional study**

Running title

Intracerebral hemorrhage in cerebral small vessel disease

**Author(s):** Yuan Gao^1,#^, Ce Zong^1,#^, Hongbing Liu^1^,Ke Zhang^1^, Hongxun Yang^1^, Yunchao Wang^1^, Yusheng Li^1^, Bo Song^1^ , Yuming Xu^1,*^

^1^Department of Neurology, the First Affiliated Hospital of Zhengzhou University, Zhengzhou, Henan, China

**#These authors contributed equally to this article**

**Table S1. Comparison of clinical characteristics between included and excluded data.**

|  | **Excluded data** | **Included data** | **p.overall** |
| --- | --- | --- | --- |
|  | **(N=318 )** | **( N=414 )** |  |
| Sex, n(%) |  |  | 0.308 |
| Male | 188 (59.1%) | 261 (63.0%) |  |
| Female | 130 (40.9%) | 153 (37.0%) |  |
| age, year^#^ | 61.7 (10.0) | 62.5 (10.4) | 0.329 |
| WBC, x10^9^L^#^ | 6.28 (2.01) | 6.70 (2.17) | 0.006 |
| PLT, x10^9^L^#^ | 204 (74.6) | 215 (63.5) | 0.040 |
| ALB, g/L^#^ | 43.3 (20.4) | 41.6 (3.94) | 0.162 |
| SBP, mmHg^#^ | 141 (18.6) | 141 (18.3) | 0.736 |
| DBP, mmHg^#^ | 86.3 (30.5) | 83.8 (11.7) | 0.162 |
| Hypertension, n(%) |  |  | 0.319 |
| No | 51 (16.4%) | 81 (19.6%) |  |
| Yes | 260 (83.6%) | 333 (80.4%) |  |
| CHD, n(%) |  |  | 0.125 |
| No | 273 (85.8%) | 371 (89.6%) |  |
| Yes | 45 (14.2%) | 43(10.4%) |  |
| CVD, n(%) |  |  | 0.312 |
| No | 161 (56.9%) | 249 (60.7%) |  |
| Yes | 122 (43.1%) | 161 (39.3%) |  |
| Smoking, n(%) |  |  | 0.127 |
| No | 213 (67.0%) | 300 (72.5%) |  |
| Yes | 105 (33.0%) | 114 (27.5%) |  |
| Drinking, n(%) |  |  | 0.643 |
| No | 233 (73.3%) | 310 (74.9%) |  |
| Yes | 85 (26.7%) | 104 (25.1%) |  |
| FBG, mmol/L^#^ | 5.59 (1.84) | 5.98 (2.21) | 0.010 |
| HbA1c, %^#^ | 6.47 (1.30) | 6.45 (1.81) | 0.878 |
| Homocysteine, μmol/L^#^ | 17.3 (14.6) | 16.4 (11.3) | 0.381 |
| TC, mmol/L^#^ | 5.64 (28.2) | 3.98 (1.13) | 0.298 |
| TG, mmol/L^#^ | 1.59 (1.16) | 1.45 (1.10) | 0.108 |
| HDL, mmol/L^#^ | 1.14 (0.28) | 1.12 (0.32) | 0.422 |
| LDL, mmol/L^#^ | 2.47 (0.93) | 2.45 (0.96) | 0.776 |

*:median (IQR);#: mean± standard deviation

**Abbreviations:** SBP, systolic blood pressure; DBP, diastolic blood pressure; CHD, coronary heart disease; CVD, cerebrovascular disease; WBC, white blood cell; PLT, platelet; ALB, albumin; FBG, fasting blood glucose; HbA1c, Glycation Hemoglobin; TC, Total cholesterol; TG, Triglycerides; LDL, low-density lipoprotein; HDL, high-density lipoprotein

- **Fig. S1.** Proportion of cerebral hemorrhage in different locations.

- **Fig S2.** Distribution of different numbers of cerebral hemorrhage
